# Selumetinib suppresses cell proliferation, migration and trigger apoptosis, G1 arrest in triple-negative breast cancer cells

**DOI:** 10.1186/s12885-016-2773-4

**Published:** 2016-10-21

**Authors:** Yan Zhou, Shuchen Lin, Kuo-Fu Tseng, Kun Han, Yaling Wang, Zhi-hua Gan, Da-liu Min, Hai-yan Hu

**Affiliations:** 1 Department of Oncology, Shanghai Jiao Tong University Affiliated Sixth People’s Hospital, Shanghai, 200233 China; 2 Biophysics Department of Oregan State University, ALS-2139, Corvallis, OR 97330 USA

**Keywords:** Selumetinib, Triple-negative breast cancer, miR-302a, CUL1

## Abstract

**Background:**

Triple-negative breast cancer (TNBC) has aggressive progression with poor prognosis and ineffective treatments. Selumetinib is an allosteric, ATP-noncompetitive inhibitor of MEK1/2, which has benn known as effective antineoplastic drugs for several malignant tumors. We hypothesized that Selumetinib might be potential drug for TNBC and explore the mechanism.

**Methods:**

After treated with Selumetinib, the viability and mobility of HCC1937 and MDA-MB-231 were detected by MTT, tunnel, wound-healing assay, transwell assay and FCM methods. MiR array was used to analysis the change of miRs. We predicted and verified CUL1 is the target of miR-302a using Luciferase reporter assay. We also silenced the CUL1 by siRNA, to clarify whether CUL1 take part in the cell proliferation, migration and regulated its substrate TIMP1 and TRAF2. Moreover, after transfection, the antagomir of miR-302a and CUL1 over-expressed plasmid into HCC1937 and MDA-MB-231 cell accompanied with the Selumetinib treatment, we detected the proliferation and migration again.

**Results:**

Selumetinib reduce the proliferation, migration, triggered apoptosis and G1 arrest in TNBC cell lines. In this process, the miR-302a was up-regulated and inhibited the CUL1 expression. The later negatively regulated the TIMP1 and TRAF2. As soon as we knockdown miR-302a and over-expression CUL1 in TNBC cells, the cytotoxicity of Selumetinib was reversed.

**Conclusions:**

MiR-302a targeted regulated the CUL1 expression and mediated the Selumetinib-induced cytotoxicity of triple-negative breast cancer.

## Mini-Abstract

Selumetinib inhibited the proliferation and migration of TNBC cell.

## Background

Breast cancer is one of the most common cancer deaths in female. Estrogen receptor(ER)-negative breast cancer constitutes approximately 30 % of breast cancer cases. Triple-negative is defined as a subgroup with ER, PR(progesterone receptor) and human epidermal growth factor receptor 2 (HER2) all negative. TNBC are assumed importance for its molecular characters, aggressive progress and distinct tranfer ability [1, 2]. Beneficial results of current anti-HER2 or hormonal therapy could not improve the curative effect of chemotherapy. In the absence of proper treatments, TNBC often progresses to metastatic lesions in the brain and lung in three years. Once being with metastasis, the 5-year survival rate of TNBC would be less than 30 %. Newly therapies are urgently needed to improve the prognosis for TNBC patients. Actually, TNBCs exhibit a high level of molecular heterogeneity without high-frequency driver mutations. About 60–70 % of TNBCs has mutations of p53. For PIK3CA mutations, it would be 11 %. No other mutations were believed as highly prevalent driver in TNBCs, which hampered the development of targeted therapy for TNBCs. So far, some new regimes such as anti-androgens, anti-mitotic, PI3KCA pathway inhibitors and so on, had been tested in TNBC. Here, we focus on the key survival pathway, mitogen-activated and extracellular signal-regulated kinase kinase (MEK)/extracellular signal-regulated kinase (ERK), which modulated by epidermal growth factor receptor(EGFR) [3, 4]. Over-expression of the EGFR is one of the key pathway regulating the proliferation and survive of cells. Hence, these genes may be good choose as therapeutic targets for TNBC [5]. Infante performed a phase Ib clinical study to determine the safety, tolerability, clinical activity and steady-state pharmacokinetics of trametinib, an oral MEK inhibitor, in combination with gemcitabine on breast cancer [6]. The results showed that trametinib combined with gemcitabine is safe and effective. Selumetinib, the benzimidazole ARRY-142886, has been reported to be highly potent MEK inhibitor, with an IC_50_ of 12 nmol/L against purified MEK [7]. There were many phase I and phase II clinical studies about Selumetinib on melanoma, colorectal cancer (CRC), non-small-cell lung cancer (NSCLC) and others, for its favorable toxicity [8–10]. Selumetinib also produces clinically meaningful increases in iodine uptake and retention in a subgroup of patients with radioiodine-refractory thyroid cancer [11]. We considered that Selumetinib might be potential drug for preventing TNBC metastasis and recurrence in a preclinical setting.

In the current study, we first found that Selumetinib inhibited proliferation and migration in two triple-negative breast cancer cell lines. Then we investigated its probable mechanism of action. MicroRNAs (miRNAs) [12] , 20–22 bp non-coding RNA, had been the hot are of cancer research for its post-transcriptional regulation function, which invovled a wide variety of biological processes, such as proliferation, differentiation, apoptosis, cell cycle and so on [13]. So far many miRs had been reported took part in MEK/ERK signaling pathway, including miR-768-3p [14], miR-221 [15], miR-199a/b-3p [16], and so on. Here, we treated TNBC cell lines HCC1937 and MDA-MB-231 with Selumetinib, and our founding indicated miR-302a/CUL1 maybe one significant downstream factors. The miR-302-367 cluster is over-expressed in embryonic stem and some kinds of carcinoma cells [17]. It works as anti-oncogene in many kinds of tumor cells, its overexpression could be of therapeutic value [18]. Kaid show that miR-302a regulated cell proliferation and self-renewal of esophageal cancer stem-like cells [19]. Here we found after treated with Selumetinib miR-302a, an well known ‘*bona fide*’ tumor suppressor, up-regulated markedly. It is difficult and costly to verified the gene regulatory networks. The bioinformatic methods offered the convenient to predicte possible diagram between miRNAs and their targets. The most publiced programs are TargetScan, miRanda, Tarbase, miRecords, RNAhybrid, and so on [20–23]. Here we found CUL-1 is the directly target of miR-302a. CUL-1 is a essential components of the p19(SKP1)/p45(SKP2)/CUL-1 complex, named SCF, as the scaffold element [24]. Previous researches suggest that SCF as ubiquitin ligase is the key factor to cell cycle and survival. Aberrant expression of CUL-1 is critical for tumorigenesis, such as lung cancer [25], gastric cancer [26]. In the present study, we found CUL-1 also demonstrate an oncogenic activity of the OS.

## Methods

### Cell culture and cell proliferation assay

After planted in 96- or 6-well plates (Corning, USA) using DMEM with 10 % fetal bovine serum (FBS) at 37 °C in humidified 5 % CO_2_, HCC1937 and MDA-MB-231 cells were exposed for 24 h to various doses of Selumetinib(Sigma-Aldrich, Louis, MO). For the transfected process, cells were with starved in DMEM without FBS for 6 h, then miR-302a-AMO, miR-302a-MIMIC, NC or pcDNA3.1-CUL1 were added with Lipofectamine 2000 Reagent (Invitrogen) following the manufacturer’s protocol. Cell proliferation assays were performed with tetrazolium salt (MTT) array according to the manufacturer’s protocol.

### Evaluation of cell apoptosis by tunnel and FCM

For FCM detection, the procedures were same as the cell culture previously. All cells of each group were collected and stained with Annexin V/PI following the instruction(BioVision, Palo Alto, CA, USA) The resulting was analyzed using CellQuest software (Becton Dickinson, San Jose, CA).

For tunnel test, the conditions were little different. First, cells were cultured with or without IC50 Selumetinib. For the rescue test, we first transferred the miR-302a AMO or pcDNA3.1-CUL1 or the negative control for 6 h, then we changed the medium which contain the 10 μM Selumetinib. Visualized apoptotic cells were labeled with the In Situ Cell Death Detection kit (Roche) to detected positive ratio of terminal deoxytransferase-mediated dUTP-biotin nick end labelling (TUNEL) following to the manufacturer’s recommendations.

### Cell cycle analysis

The procedure was the same as that described previously for cell culture. After washed with phosphate-buffered saline (PBS), all cells were fixed with 70 % ethanol at −20 °C for 24 h. Then washed the cell with PBS/1 % BSA again, stained with 30 μg/ml propidium iodide containing 0.25 mg/ml RNase A for 0.5 h in the dark, and calculated the cell cycle process ratio by FCM using Cell FIT software(Becton Dickinson, San Jose, CA).

### Wound-healing assay

In order to evaluated the motility change of TNBC cells with Selumetinib, wound healing/scratch assay was performed. TNBCs were seeded in six-well plates overnight, then scraped the confluent cell monolayer using a 200 μL sterile pipette tip. After wased with PBS twice then cultured with new DMEM medium (including 10 % FBS with or without IC50 Selumetinib). For the rescue test, we first transferred the miR-302a ASO or pcDNA3.1-CUL1 or the negative control for 6 h, then we made the wound. The newly mediums contain the 10 μM Selumetinib. 48 h later, photo images of the plates were photographed.

### Cell migration assay

For the migration assay, 1.0 × 10^5^ HCC-1937 or MDA-MB-231 cells were seeded in 24-well transwell insert (pore size 8 μm; Corning, Inc., Corning, NY). The culture conditions were equal to above. After incubated for 12 h, the cells adhering in the lower layer of insert were fixed and stained with 0.1 % crystal violet. Photographed under light microscope at 200 × magnification.

### Detection of differentially expressed miRNAs by miRNA microarray

HCC-1937 cells treated with or without Selumetinib at its IC50 for 24 h were harvested and subsequently analyzed using a miRNA microarray (Kangcheng Biotech Company, Shanghai, China). Briefly, total miRNA was labeled, hybridized according to manufacturer’s protocol. The slides were scanned by an Axon GenePix 4000B microarray scanner. Data filtering, log2 transformation, and miR normalization hot map were provided by Kangcheng Com.

### Targeted in vitro luciferase reporter assay

Luciferase reporter assay using the psi-Check2 plasmid was performed as described previously to detected the interact between the miR and target [27]. The sequences used to create the wild Check2-CUL1 constructs were as follows: forward 5′-**AACTCGAG**GACCCGCAGCAAATAGTTCA-3′ (***XhoI*** site in bold) and reverse 5′ **AATGCGGCCGC**CAATGTTCAGCGTAACCCAA-3′ (***NotI*** site in bold).

### Quantitative real-time PCR of miR-302a and CUL1 expression

Total RNA of each group were abstracted with Trizol. The primer of miR-302a was purchased from Jima Com(Shanghai, China). The primers of CUL1 and its substrates TIMP and TRAF2 were as follow. The QRT-PCR method was performed as described previously to detected the interact between the miR and target [27].

**Table:** 

	Forward	Reverse
CUL1	5'-GCGAGGTCCTCACTCAGC-3'	5'-TTCTTTCTCAATTAGAATGTCAATGC-3'
TIMP	5'-GCCATGGAGAGTGTCTGCGGATACTTCC-3'	5'-GCCACGAAACTGCAGGTAGTGCTGT-3'
TRAF2	5'GACCAGGACAAGATTGAGGC-3'	5'-GCACATAGGAATTCTTGGCC-3'
GAPDH	5'-GAAGGTGAAGGTCGGAGT-3'	5'-GAAGATGGTGATGGGATTTC-3'

### Western blot analysis

The total protein were lysed in RIPA buffer and extracted. 10 % SDS polyacrylamide gel was used to separated the proteins. After blocking with 5 % fat-free milk for 1 h, the membranes were incubated with antbody of CUL1 (mouse monoclonal; Invitrogen, USA), TIMP (Rabbit monoclonal; Cell Signaling Technology, MA) or TRAF2 (Rabbit polyclonal, Abcam, USA) overnight at 4 °C. Blots were washed with PBST and incubated with the secondary antibody for 1 h. Took the photo using enhanced chemiluminescence.

### siRNA targeting CUL1

Designed and synthetized siRNA-CUL1(5′-CUAGAUACAAGAUUAUACAUGCGG-3′) or the control GAPDH-siRNA from GenePharma Com(Shanghai, China). The full-length CUL-1 .

### Construction of the CUL1 plasmid

The CUL-1 gene was cloned into pcDNA3.1 plasmid using the primer of CUL-1 sense 5′-CAGGATCCCGTCAACCCGGAGCCAGA-3′ (BamHI site in bold) and antisense 5′-AAGCGGCCGCAGAAGGGWAGCCMG-3′ (NotI site in bold).

## Results

### Selumetinib inhibited proliferation and migration in TNBC cells

**Fig. 1:**
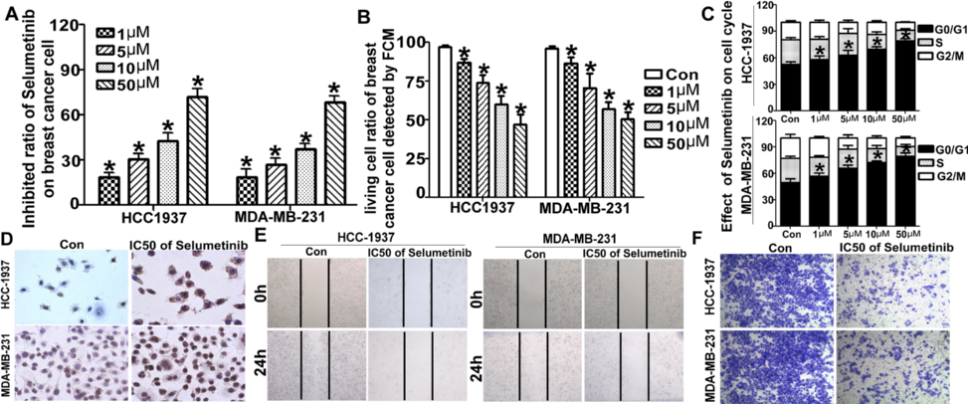
Selumetinib regulates apoptosis and the cell cycle in breast cancer cells. **a** Selumetinib inhibited the viability of TNBC. After exposure to various concentration (from 1 to 50 μM) of Selumetinib for 24 h, the proliferation inhibited ratios of HCC-1937 and MDA-MB-231 were determined using the MTT assay. The formula is Inhibition ratio = (1- Experimental OD / Control OD)*100 %. For the untreated control group, the inhibition ratio is 0(For HCC1973 cells, the inhibition ratios are 18.53 ± 5.75, 30.57 ± 6.89, 42.83 ± .89, 42.8ition ratios are 18.53n For MDA-MB-231 cells, the inhibition ratios are 17.83 ± 8.43, 27.27 ± 7.41, 37.57 ± 5.65 and 68.53 ± 7.71 respectively. *P < 0.001 compared with the untreated control group.). **b** HCC-1937 and MDA-MB-231 cells were treated with 1–50 μM Selumetinib for 24 h. It showed statistical analysis of the living cell ratio using Annexin V and PI stain by FASC method. The living cell is the double negative cells in the third quadrant (For HCC1973 cells, the living cells ratios are 86.67 ± 4.51, 73.67 ± 9.07, 59.93 ± 9.46 and 47.03 ± 10.57 respectively. For MDA-MB-231 cells, the living cells ratios are 86.23 ± 7.29, 70.53 ± 15.74, 56.73 ± 7.94 and 50.13 ± 8.48 respectively. *P < 0.01 compared with the untreated control group) **c** HCC-1937 and MDA-MB-231 cells were treated as **b**. The cells were stained with PI only, and the cell cycle distribution was determined using FACS too. The statistical analysis show the cells were arrest in G1 stage (For HCC1973 cells, the G1 ratios are 57.03 ± 5.93, 62.39 ± 7.44, 67.21 ± 1.92 and 77.69 ± 2.21 respectively vs 48.277vely. For MDA-MB-231 cells, the G1 ratios are 55.29 ± 3.66, 65.27 ± 2.84, 70.33 ± 1.06 and 75.84 ± 2.92 respectively vs 47.16 ± 4.07. *P < 0.01 compared with the untreated control group). **d** Tunnel method was used to detect the apoptosis too. In Selumetinib group, there were brown particles in positive cell. **e** Wound-healing assay showed untreated cell rapidly closed the scratch wounds compared with the IC50 dose of Selumetinib. **f** Transwell migration assays indicated that the Selumetinib resulted in significant reduction of cell migration

Selumetinib has shown the particularly exciting therapeutic effect on many kinds of cancer. Cell proliferation was assessed in HCC1937 and MDA-MB-231 cells. Selumetinib reduced the viability ratio of both two TNBCs in dose-dependent manner (Fig. 1a). The IC50 of Selumetinib for HCC1937 and MDA-MB-231 were 15.65 and 12.94 respectively. Apoptosis and cell cycle arrest are the main reason for the inhibition of cell growth. Here we found Selumetinib trigged apoptosis and arrest of G1 stage in dose-dependent manner too (Fig. 1b, c and d). Moreover, we explored the effect of Selumetinib on cell mobility. Compared with the control group, TNBCs with IC50 of Selumetinib slowly closed the scratch wounds (Fig. 1e). he Fig. 1f showed that Selumetinib treatment led to significantly decreased in cell migration ability than the untreated control cells.

### Selumetinib up-regulated miR-302a and down-regulated CUL1 expression

**Fig. 2:**
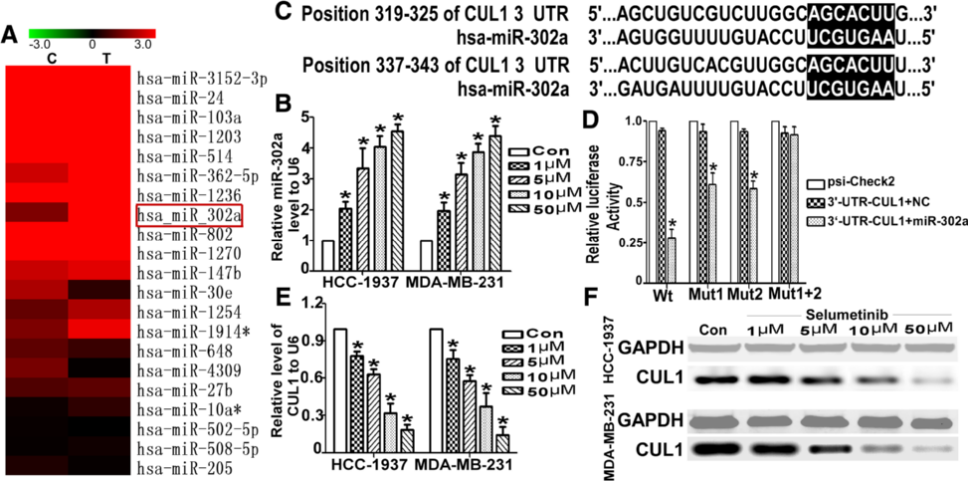
Selumetinib up-regulates miR-302a and down-regulated CUL1 in TNBC cells. **a** Microarray analysis was used to compare the expression profiles of 703 miRNAs in HCC-1937 cells that were untreated or treated with Selumetinib. MiR-302a, one of most markedly up-regulated miRs, is labeled with a red box. **b** As detected by qRT-PCR, the miR-302a levels were dramatically increased by approximately 2- to 5-fold in the dose-dependent manner in Selumetinib groups compared with the untreated group. (For HCC1973 cells, the miR-302 levels are 2.03 ± 0.41, 3.33 ± 1.12, 4.03 ± 0.61 and 4.53 ± 0.41 respectively. For MDA-MB-231 cells, the miR-302 levels are 1.97 ± 0.47, 3.13 ± 0.67, 3.87 ± 0.47 and 4.41 ± 0.56 respectively.*P < 0.01 compared with the untreated control group, which is 1.). **c** The selection criteria of the miRNA targets were based on their common detection in the target prediction online databases as well as the full complementarity between the seed region of miR-302a and the 3′UTR of CUL1. **d** HEK 293 cells were co-transfected with miR-302a-MIMIC, psi-Check2, WT-psi-Check2-CUL1 or MUT-psi-Check2-CUL1. The luciferase activity levels were measured 24 h after transfection. The results from at least three independent experiments are presented as the means ± SE. In this panel, the luciferase assay results show the regulation of CUL1 by miR-302a (For Wt group, after transfect the miR-302 the luciferase activity was only 0.27 ± 0.05. As for mutated the first seed sequence, the luciferase activity was 0.62 ± 0.07. As for mutated the second seed sequence, luciferase activity was 0.57 ± 0.04. *P < 0.01 compared with the control group, which is 1.). **e** The CUL1 mRNA level was reduced with the exposed under Selumetinib in dose-dependent manner (For HCC1973 cells, the CUL1 levels are 0.81 ± 0.04, 0.63 ± 0.08, 0.43 ± 0.14 and 0.23 ± 0.07 respectively. For MDA-MB-231 cells, the CUL1 levels are 0.78 ± 0.07, 0.64 ± 0.11, 0.37 ± 0.07 and 0.24 ± 0.05 respectively.*P < 0.01 compared with the untreated control group, which is 1.). **f** The change of CUL1 protein has showed a similar trend

miRs are involved in regulating gene transcription and cell biological function. Here we detected the change of miRs in MDA-MB-231 treated with Selumetinib. miRNA array analysis showed miR-302a(sequence: GUGAAAUGUUUAGGACCACUAG) raised 3.856 times (Fig. 2a). Furthermore we verified the expression level of miR-302a was markedly and stable up-regulated in TNBCs by QRT-PCR (Fig. 2b). For miR-302a, it had been considered as a tumor suppressor [28]. A series of bioinformatics software made it easier to look for targets of miRs. miRanda show there was two combine seed sequence between miR-302a and the 3′-UTR of CUL1 (Fig. 2c). To determine whether CUL-1 is the functional target of miR-302a, we constructed WT or MUT psiR-CHECK2-CLU-1 recombinant plasmid. After transfected miR-302a-MIMIC and the plasmids in 293 T cells for 48 h, the luciferase reporter activity was detected. The luciferase activity of WT Check2-CUL1 was reduced to approximately 35–40 % of control group(*P* < 0.001 Fig. 2d). Conversely, there were no statistical difference between MUT group and control. What is the effect of Selumetinib on CUL-1, Fig. 2e and f described that Selumetinib reduced both mRNA and protein level of CUL-1 in dose-dependent manner. It is the reversely changes to miR-302a, which also proved miR-302a negative regulated CLI-1 indirectly.

### CUL1 regulated the degradation of key regulatory proteins

**Fig. 3:**
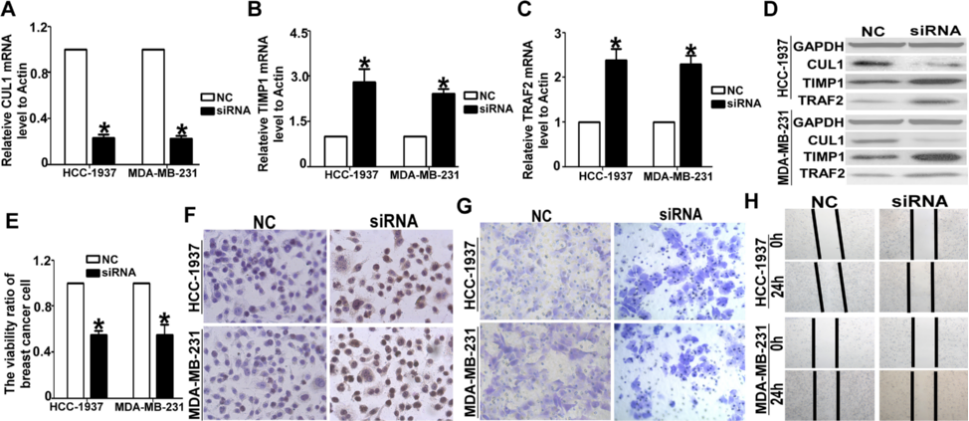
Knocked-down the CUL1 lead to the same effect of Selumetinib. **a** The siRNA could effective inhibited the CUL1 expression on mRNA levels (For HCC1973 cells, the CUL1 level was 0.23HCC19. For MDA-MB-231 cells, the CUL1 level was 0.22 ± 0.04.*P < 0.01 compared with the NC group, which is 1.). **b** After silencing the CUL1, the TIMP1 level was increased (For HCC1973 cells, the TIMP1 level was 2.81 ± 0.75. For MDA-MB-231 cells, the TIMP1 level was 2.41 ± 0.23.*P < 0.01 compared with the NC group, which is 1.). **c** The tendency of TRAF2 mRNA expression levels in MDA-MB-231 and HCC-1937 cells were equal to TIMP1 determined by QRT-PCR (For HCC1973 cells, the TRAF2 level was 2.38 ± 0.45. For MDA-MB-231 cells, the TRAF2 level was 2.29 ± 0.33. *P < 0.01 compared with the NC group, which is 1.). **d** the CUL1, TIMP1 and TRAF2 protein expression levels in HCC-1937 and MDA-MB-231 cells were examined by WB analysis. **e** After transfected the siRNA-CUL into HCC-1937 and MDA-MB-231 cells, the cell proliferation ratio was inhibited significantly (For HCC1973 cells, the viability ratio was 0.55 ± 0.06. For MDA-MB-231 cells, the viability ratio was 0.53 ± 0.15. *P < 0.01 compared with the NC group, which is 1.). **f** the Tunnel analysis clarified this negative effect of siRNA-CUL1. **g** The transwell analysis showed, the numbers of the migrated cells in the NC group of both two TNBC cells, are almost double that are in the siRNA-CUL1 group **h**. The wound-healing assay showed the cell migration ability was reduced observably in siRNA-CUL1 group. For the NC group, the gap closure rate is more than two to thirds in both HCC-1937 and MDA-MB-231 cells. For siRNA-CUL1 group, the gap closure rate is less than one thirds

In order to clarify the CUL1 play important role in Selumetinib on TNBC cells, we further detected the change of two downstream substrates of Cul-1, TIMP1 and TRAF2. First we knock down the CUL1 by siRNA. QRT-PCR and WB results show the siRNA effectively silencing the CUL1 expression and lead to TIMP1 and TRAF2 up-regulating (Fig. 3a to d). Equal to our hypothesis, as soon as the CUL1 were knocked down, the cell proliferation and migration were reduced (Fig. 3e to g).

### Adjusted miR-302a/CUL1 level reversed the effect of Selumetinib

**Fig. 4:**
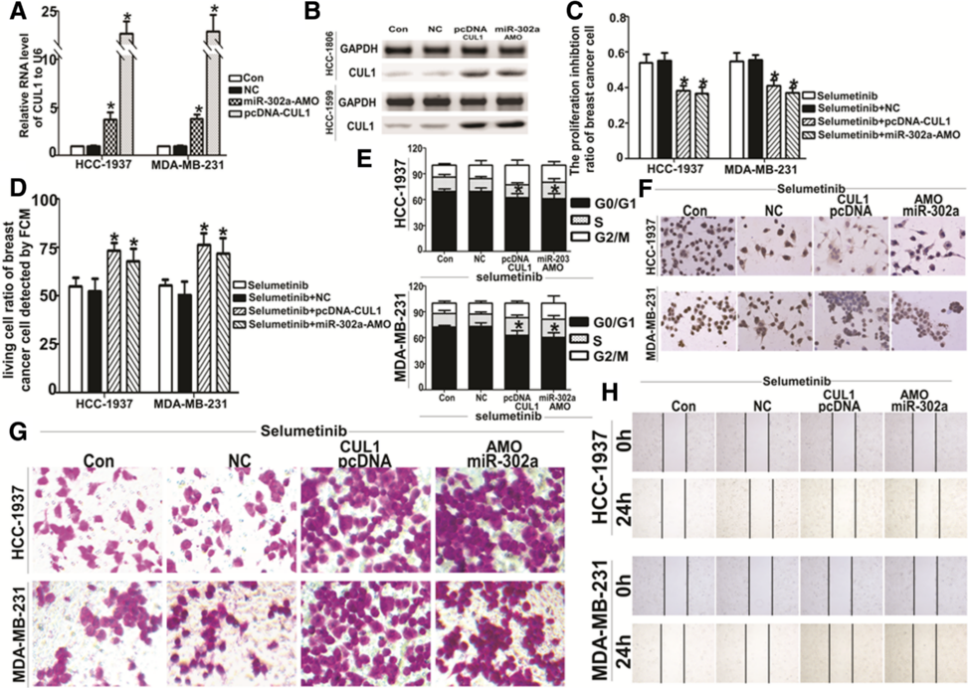
Characterization of cells transfected with miR-302a-AMO and the CUL1 expression plasmid. After transfected miR-302a-AMO or the pcDNA3.1-CUL1 plasmid in HCC-1937 and MDA-MB-231 cells for 6 h, HCC-1937 and MDA-MB-231 cells were exposed on Selumetinib (10 μM). The CUL1 mRNA (**a**) and protein (**b**) expression levels were detected by qRT-PCR and western blot analysis, respectively. The CUL-1 level on both mRNA and protein increased significantly in miR-302a-AMO and pcDNA3.1-CUL1 group (For HCC1973 cells, the CUL1 levels were 0.97 ± 0.08, 3.81 ± 1.17 and 14.18 ± 3.56 respectively. For MDA-MB-231 cells, the CUL1 levels were 0.95 ± 0.15, 3.86 ± 0.77 and 17.44 ± 4.71 respectively. *P < 0.01 compared with the Selumetinib control group, which is 1). **c** Cell viability was detected by MTT method after single or combined treatment with Selumetinib and miR-302a-AMO or the pcDNA3.1-CUL1 plasmid (For HCC1973 cells, the inhibition ratio were 53.72 ± 8.34 vs 55.07 ± 7.83, 38.17 ± 4.89 and 36.73 ± 6.11 respectively. For MDA-MB-231 cells, the inhibition ratio were 54.83 ± 7.81 vs 55.33 ± 5.43, 41.11 ± 5.47 and 37.27 ± 4.25 respectively. *P < 0.01 compared with the Selumetinib control group). **d** Change in the apoptotic ratio of was HCC-1937 and MDA-MB-231 cells were detected by FACS (For HCC1973 cells, the living cell ratio were 55.07 ± 7.61 vs 52.33 ± 11.52, 73.27 ± 7.26 and 68.03 ± 10.95 respectively. For MDA-MB-231 cells, the living cell ratio were 55.37 ± 5.51 vs 50.53 ± 11.91, 76.43 ± 10.21 and 71.82 ± 14.07 respectively. *P < 0.01 compared with the Selumetinib control group). **e** As soon as the same treatment on HCC-1937 and MDA-MB-231 cells, the cell cycle arrest was in line with apoptosis trends ((For HCC1973 cells, the G1 ratios are 69.53 ± 5.35 vs 69.02 ± 7.28, 62.33 ± 8.27 and 60.86 ± 10.69 respectively. For MDA-MB-231 cells, the G1 ratios were 72.53 ± 2.91 vs 72.94 ± 7.55, 62.53 ± 9.33 and 60.56 ± 9.33 respectively. *P < 0.01 compared with the Selumetinib control group). **f** Another apoptosis detected method, tunnel, produced the identical results. **g** and **h** indicated the negative effect of Selumetinib on migration of HCC-1937 and MDA-MB-231 were reverse by miR-302a-AMO and CUL1 over-expression plasmid

In order to more deeply investigate the miR-302a/CUL-1 pathway is relevance of Selumetinib effect. We over-expressed CUL1 with pcDNA3.1-CUL1 plasmid and inhibited miR-302a with its AMO oligonucleotide, which induced up-regulating of CUL1 on both the mRNA and protein level (Fig. 4a and b). The effect of Selumetinib on TNBCs was reversed by miR-302a-AMO and pcDNA3.1-CUL1 plasmid. For the inhibition of viability, in both HCC1937 and MDA-MB-231 cells, the miR-302a-AMO and pcDNA3.1-CUL1 group were 1.63- to 2.3-fold lower than other two control groups (Fig. 4c). The ratio of apoptosis and G1 stage were alos lower than two control groups (Fig. 4d to f). The Wound-healing assay and transwell test showed the migration inhibited effect of Selumetinib on HCC1937 and MDA-MB-231 were reverse by miR-302a-AMO and CUL1-overexpressed plasmid (Fig. 4g, h).

## Discussion

Aberrant activation of RAS/Raf/MEK/ERK signaling pathways had been reported in many kinds of cancer and been considered as targeted for its oncogenic effect. Not only pre-clinically but also many phase I or II clinical trial had been displayed the obvious therapeutic effects in solid tumor [27–29]. Some research reported that BRAF mutation was closely related to the sensitivity of Selumetinib [30]. Chen found that Selumetinib selectively rescued primary glial progenitors from TMX toxicity, such as cognitive dysfunction and changes in CNS metabolism, hippocampal volume, and brain structure, in vitro while enhancing TMX effects on MCF7 [31]. MEK pathway also plays key role in TNBC. Here, we found MAP/ERK kinase (MEK) 1/2 inhibitor, Selumetinib, repress the viability and induced apoptosis of HCC1937 and MDA-MB-231 in a dose-dependent manner. The G1 arrest and mobility declined were also linked to dose of Selumetinib (Fig. 1a to d). Then we goes deeply into the mechanism. We screened miRNA profile of MDA-MB-231 with or without Selumetinib (Fig. 2a). miR-302a was significantly and gradually up-regulated miRNAs with the concentration of Selumetinib in both MDA-MB-231 and HCC-1937 (Fig. 2b). We focus on microRNA (miR)-302 family for its tumor supperessor function in many kinds of tumor [32, 33]. Yan reported miR-302 was important to miRNA-induced pluripotent stem cells (mirPS) of endometrial cancer cell lines, take part in the inhibition of cell proliferation and tumorigenicity [34]. Previous papers revealed that miR-302a regulated the expression of AKT1 [35], NR2F2 [36], CDK2 [37] and so on targeted genes. By targetscan, we predicted there were two complementary sequences in the miR-302a and 3′UTR-CUL1 (Fig. 2c). Luciferase reporter assay showed miR-302a negative regulation of CUL1 directly (Fig. 2d). According to Fig. 2e and f, it was confirmed that CUL1 level was closed related with Selumetinib concertration. CUL1 is a key component of SCF ubiquitin ligases [38]. SCF promotes the ubiquitination and degradation of a broad range of proteins involved in cell cycle progression, signal transduction and transcription. As a key member of SCF, CUL1 is over-expressed in many kinds of cancer [39–41] and represent as target molecular for therapy [42–44]. In this paper, we found Selumetinib could inhibit both proliferation and migration in TNBC cells, and miR-302a/CUL maybe the key factor in this process. So we assume that it is equal to Selumetinib that we knocked down the CUL1 in TNBC cells. We also choose tow substrate, TIMP1 and TRAF2 of CUL1 to clarify this hypothesis. As expected, after silencing the CUL1, the viability and migration ability of TNBC cell were reduced markedly. For further prove miR-302a/CUL-1 is the operator nodes of Selumetinib onTNBCs. We regulated the miR-302 or CUL-1 level using miR-302a-AMO or CUL1 over-expression plasmid respectively in HCC1937 and MDA-MB-231 cells. Figure 4 indicated the effect of Selumetinib reversed accompany with raising of CUL-1 and silencing of miR-302a.

## Conclusion

MEK pathway has been shown over-activated in TNBC. Based on our results, MEK1/2 inhibitor, Selumetinib, reduced viability through inducing apoptosis and G1 arrest, meanwhile the inhibition of mobility by Selumetinib was also be found in TNBCs. In these processes, we indicated miR-302a/CUL1 work as critical pathway in Selumetinib on TNBC.
